# The status of TKI/acid-suppressant concomitant use in 44 hospitals in China: A cross-sectional descriptive study

**DOI:** 10.1097/MD.0000000000031770

**Published:** 2022-11-18

**Authors:** Fangting Chen, Wendong Yao, Fan Wu, Rui Xie, Jianping Wang, Zheng Shi

**Affiliations:** a Yongkang Orthopaedic Hospital (Yongkang Sixth People’s Hospital), Yongkang, China; b The First Affiliated Hospital of Zhejiang Chinese Medical University (Zhejiang Provincial Hospital of Chinese Medicine), Hangzhou, China.

**Keywords:** acid-suppressant, irrational combination, pharmacoeconomic indicators, prescription, rational intervention, survey, tyrosine kinase inhibitors

## Abstract

The irrational use of tyrosine kinase inhibitors (TKIs) has attracted increasing attention, especially because of drug–drug interactions. The objective of this study was to analyze TKI prescriptions and evaluate the rationality of concomitant use of TKIs and acid-suppressants. TKI prescriptions from 2016 to 2018 were collected from hospitals in Beijing, Guangzhou, Hangzhou, and Zhengzhou for 40 d/yr. Focusing on the data in 2018, we analyzed the pharmacoeconomic indicators of TKIs and the number and proportion of different coprescriptions. The evaluation criteria for coprescriptions were based on clinical literature and package inserts. A total of 41,738 TKI prescriptions were assessed. The total dose and sales of imatinib were the highest, the medication days and defined daily doses of gefitinib were the highest, and the highest defined daily cost was sunitinib. Meanwhile, there were 17 TKIs with drug utilization indices of ≤ 1.0. The irrational combination rate of prescriptions of non-cancer-related departments was high in 3 cities, but not Hangzhou. The irrational combination rate of prescription of inpatient prescriptions was > 23% in the 4 cities. The combined use of TKIs and acid-suppressants is common in China and may have a clear or potential impact on the pharmacokinetics, pharmacodynamics, and adverse drug reactions of TKIs. Therefore, it is urgent to implement necessary interventions to stop such irrational use or if the combined use is necessary, to correct adverse consequences. The aims should be to achieve safe and effective use of TKIs and reduce unnecessary costs.

## 1. Introduction

Currently, tyrosine kinase inhibitors (TKIs) are the most common oral chemotherapy drugs used in tumor therapy.^[1–4]^ As of March 1, 2019, 35 TKI preparations have been approved by the Food and Drug Administration, all of which are orally effective.^[5]^ Most TKIs have been approved for use in China and have gradually been included in the medical insurance list in recent years. Therefore, TKIs are increasingly used in China for tumor treatment with definite clinical effects and affordable costs.^[6–9]^

As oral chemotherapy drugs, TKIs increase the flexibility and convenience of patients’ self-medication, but also increase the possibility of potential drug–drug interactions (DDIs).^[10–14]^At present, all marketed TKIs have different degrees of gastrointestinal adverse reactions (according to the package inserts of brand name drugs), hence some patients suffer from gastrointestinal diseases; therefore, combined prescriptions of TKIs and acid-suppressants are often used habitually, and in some cases they may be required. The main acid suppressants used in clinics are proton pump inhibitors (PPIs) and histamine 2-receptor antagonists (H2RAs).

Because the absorption of most TKIs mainly depends on the acidic environment in the stomach, combined TKI/acid-suppressant use will affect absorption to varying degrees.^[15–26]^ In addition, some acid suppressants themselves are inhibitors or agonists of microsomal enzymes, which also have different effects on the metabolism of TKIs; these include cimetidine, famotidine, esomeprazole, omeprazole, and lansoprazole. The concomitant use of TKIs and acid suppressants may affect pharmacodynamics (PD) or even adverse drug reactions (ADRs) because of the impact of acid suppressants on the pharmacokinetics (PK) of TKIs. Therefore, the purpose of this study was to analyze the rationality and impact of coprescriptions on TKI treatment by evaluating the current situation in Grade III Class A hospitals in China to provide a basis for rational intervention.

## 2. Methods

### 2.1. Data collection, inclusion criteria, and evaluation standard

The following information was collected: city, time, hospital code, prescription code, clinical department, patient source, drug commodity name, drug generic name, drug specification, medication route, total amount and price of medicine taken, usage, and single dosage. Data were obtained from the Hospital Prescription Cooperate on Project in China, whose main purpose is to analyze and study the current situation and developing trends of the use of hospital drugs in clinical departments nationwide through a unified and scientific sampling method.^[15]^ More than 100 hospitals from 9 cities participated in the project, providing the research group with data on prescriptions for each sample day. There were 40 days of sampling every year, 10 days of sampling every quarter, and 3–4 days of random sampling every month (except weekends and holidays).^[27]^ This study was performed according to the guidelines of the World Medical Association and the Declaration of Helsinki. The ethics committee of our hospital’s institutional review board approved this survey.

The 44 hospitals included in the study were Grade-III class-A general hospitals from 4 major cities (Beijing, Guangzhou, Hangzhou, and Zhengzhou) located in the north, south, east, and west of China. Therefore, the data on TKI prescriptions were representative nationwide. We collected TKI prescription data for all outpatients and inpatients within 40 days of each year, from 2016 to 2018.

The World Health Organization recommends a defined daily dose value for TKIs. All pharmacoeconomic indicators (defined daily dose system [DDD], defined daily cost [DDC], and drug utilization index [DUI]) of TKIs were calculated and analyzed according to their definitions. The evaluation criteria for combined TKI/acid-suppressant use were based on the drug clinical literature and the package inserts of brand name drugs. The formulas for DDD, DDC, and DUI calculating were shown as following:


$$\begin{array}{l} DDD\;=\;Total\;doses/\;Defined\;daily\;dose \end{array}$$

$$\begin{array}{l} DDC\;=\;Total\;sales/\;DDD \end{array}$$

$$\begin{array}{l} DUI\;=\;DDD/Medication\;days \end{array}$$

We have regarded the prescriptions with clear evidence of DDI and potential influence as irrational combination prescriptions, and have eliminated the combination prescriptions with no influence or dispute. As far affected PK, clear evidence of DDI included significant effects, slight effects, effects but extent not mentioned. In PK, clear evidence of DDI included significant effects and effects but extent not mentioned. As for PK, clear evidence of DDI included significantly affecting the incidence of ADRs and aggravating ADRs symptoms but not affecting the incidence.

### 2.2. Statistical analysis

The prescription information was processed using Microsoft Access software and exported to Microsoft Office Excel® 2017 (Microsoft Corp., Redmond, WA) for statistical analysis.

## 3. Results

In total, 41738 TKI prescriptions were dispensed during 2016–2018. Eighteen kinds of TKI were used in these hospitals in 2018, including afatinib, apatinib, axitinib, icotinib, anlotinib, osimertinib, dasatinib, erlotinib, gefitinib, crizotinib, lapatinib, nilotinib, regorafenib, seritinib, sunitinib, sorafenib, ibrutinib, and imatinib, all of which were oral preparations. The proportion of TKI prescriptions was highest in Guangzhou (41.88%), followed by Zhengzhou and Beijing, and lowest in Hangzhou (14.87%).

### 3.1. TKI usage trend

During 2016–2018, the use of TKIs (Table 1) in the 4 cities showed a rapid growth trend. In 2017, the types, prescriptions, quantity, and amount of TKIs used increased by 11.1%, 48.1%, 39.8%, and 5.8%, respectively, compared with 2016; in 2018, they increased by 70%, 80.8%, 71.1%, and 40.4%, respectively, compared with 2017.

**Table 1:** Changes in the types, prescriptions, quantity, and cost of TKIs used from 2016 to 2018.

Year	Types of TKI	Growth rate (%)	Prescriptions of TKI	Growth rate (%)	Quantity of TKI	Growth rate (%)	Amount of TKI (dollar)	Growth rate (%)
2016	9	/	8092	/	444,611	/	11,801,391	/
2017	10	11.1	11,984	48.1	621,495	39.8	12,484,680	5.8
2018	18	80.0	21,662	80.8	1063,271	71.1	17,528,590	40.4

TKI: tyrosine kinase inhibitors.

### 3.2. DDD, DDC, and DUI values of different TKI

We analyzed the DDD, DDC, and DUI values of the different TKIs used in 2018. Table 2 shows that the DDD value of gefitinib and the DDC value of sunitinib were the highest, while the lowest DDD value was obtained for seritinib, and the minimum value of DDC was obtained for regorafenib. The highest DUI value was obtained for Regorafenib (5.3), and the DUI values of 17 TKIs were ≤ 1.0, including afatinib, apatinib, axitinib, icotinib, anlotinib, osimertinib, dasatinib, erlotinib, gefitinib, crizotinib, lapatinib, nilotinib, seritinib, sunitinib, sorafenib, ibrutinib, and imatinib.

**Table 2:** DDD, DDC, and DUI values for different TKIs in 2018.

TKI	Total doses (g)	Medication days (d)	Total sales (dollar)	Defined daily dose (mg)	DDD	DDC (dollar)	DUI
Afatinib	24.1	649	17,464	40	602	29.0	0.9
Apatinib	21,223.1	42,054.4	1618,997	850	24,968	64.8	0.6
Axitinib	5.9	579	34,928	10	591	59.1	1.0
Icotinib	30,231.5	87,049.2	2461,108	375	80,617	30.5	0.9
Anlotinib	20.5	1733.4	120,960	12	1709	70.8	1.0
Osimertinib	409.0	5162	640,203	80	5112	125.2	1.0
Dasatinib	1901.1	20,791.8	374,491	100	19,011	19.7	0.9
Erlotinib	2862.7	19,810	524,278	150	19,084	27.5	1.0
Gefitinib	27,028.3	106,888	3277,662	250	108,113	30.3	1.0
Crizotinib	631.5	1568	115,535	500	1263	91.5	0.8
Lapatinib	14,119.8	14,033	562,587	1250	11,296	49.8	0.8
Nilotinib	2400.0	4110	489,432	600	4000	122.4	1.0
Regorafenib	84.8	100	8480	160	530	16.0	5.3
Seritinib	45.0	100	8480	450	100	84.8	1.0
Sunitinib	46.5	1053.7	201,643	50	930	216.9	0.9
Sorafenib	20,636.6	34,609	2944,601	800	25,796	114.2	0.7
Ibrutinib	250.3	891.5	52,751	560	447	118.0	0.5
Imatinib	37,195.0	89,105.1	4000,770	400	92,988	43.0	1.0

DDC *=* defined daily cost, DDD *=* defined daily dose system, DUI *=* drug utilization index, TKI *=* tyrosine kinase inhibitors.

### 3.3. Usage of TKIs and combination prescriptions

**Figure 1.**
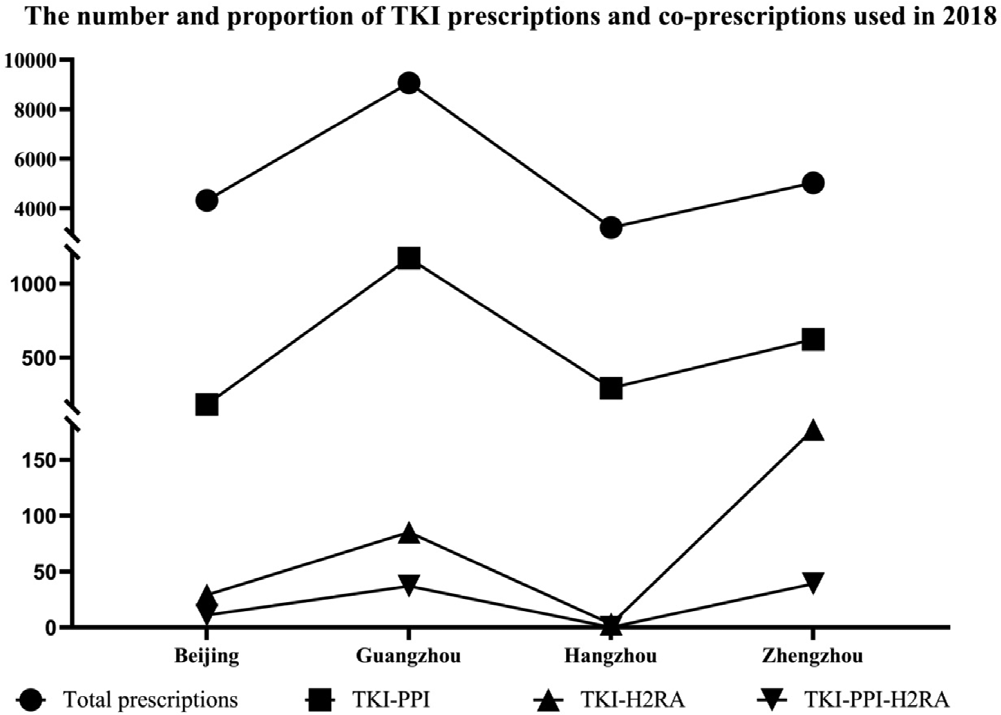
The number and proportion of TKI prescriptions and co-prescriptions used in 2018. H2RA = histamine 2-receptor antagonists, PPI = proton pump inhibitors, TKI = tyrosine kinase inhibitors.

Based on the number and proportion of TKIs and their combination prescriptions (Fig. 1), we evaluated their use in different cities in 2018. The city with the largest number of TKI prescriptions was Guangzhou (41.91%), while Hangzhou (14.89%) was the lowest; the city with the most prescriptions of TKI/PPI combinations was Guangzhou (51.56%), while Beijing (8.09%) was the lowest; the city with the most prescriptions of TKI/H2RA combinations was Zhengzhou (60.20%), while Hangzhou (1.02%) was the lowest; the prescriptions of TKI/PPI/H2RA combinations was the most in Zhengzhou (44.83%), and the least in Hangzhou (0%).

### 3.4. Influence of TKI/acid suppressant use on PK, PD, and ADR of TKIs

Based on the clinical literature and package inserts, 63.4% of the prescriptions of TKI/acid-suppressant combinations had potential effects on the PK of the TKI (no relevant research, no hint in the package inserts), while 16.8% had significant effects, 10.2% had slight effects, 9.1% had effects but extent not mentioned, and 0.5% had no effects (Table 3).

**Table 3:** Influence of TKI/acid-suppressant use on PK of TKIs in 2018.

Cities	Significant effects	Slight effects	Effects but extent not mentioned	Potential effects	No effects	Sum.
Beijing	29 (1.2%)	41 (1.7%)	14 (0.6%)	117 (4.7%)	1 (0.0%)	202(8.1%)
Guangzhou	161 (6.5%)	112 (4.5%)	131 (5.3%)	807 (32.5%)	10 (0.4%)	1221 (49.2%)
Hangzhou	47 (1.9%)	12 (0.5%)	35 (1.4%)	203 (8.2%)	0 (0.0%)	297 (12.0%)
Zhengzhou	179 (7.2%)	87 (3.5%)	47 (1.9%)	446 (18.0%)	3 (0.1%)	762 (30.7%)
Sum.	416 (16.8%)	252 (10.2%)	227 (9.1%)	1573 (63.4%)	14 (0.5%)	2482 (100.0%)
Specific TKI in co-prescriptions	Dasatinib [3-4], Erlotinib [5], Gefitinib [6]	Axitinib [7], Nilotinib [8], Seritinib [9], Ibrutinib [10], Imatinib [11]	Sunitinib [12], Sorafenib [13]	Afatinib, Apatinib, Icotinib, Anlotinib, Lapatinib, Regorafenib	Osimertinib [14], Crizotinib*	Specific TKI in co-prescriptions

PK *=* pharmacokinetics, TKI *=* tyrosine kinase inhibitor.

* According to package inserts; Significant effects: *P* < .01; Slight effects: *P* < .05; potential effects: no relevant research, no hint in the package inserts.

Regarding the effect of TKI/acid-suppressant combinations on the PD of the TKI, 79.0% of the prescriptions had potential effects, while 9.5% were without effect or meaningless influence, 7.8% had significant effects, 3.4% had effects but extent not mentioned, and 0.2% were controversial (Table 4).

**Table 4:** Influence of TKI/acid-suppressant use on PD of TKIs in 2018.

Cities	Significant effects	Effects but extent not mentioned	Potential effects	No effects or influence on clinical meaninglessness	Controversy	Sum.
Beijing	24 (1.0%)	0 (0.0%)	162 (6.5%)	16 (0.6%)	0 (0.0%)	202 (8.1%)
Guangzhou	69 (2.8%)	66 (2.7%)	948 (38.2%)	138 (5.6%)	0 (0.0%)	1221 (49.2%)
Hangzhou	47 (1.9%)	0 (0.0%)	212 (8.5%)	38 (1.5%)	0 (0.0%)	297 (12.0%)
Zhengzhou	54 (2.2%)	19 (0.8%)	638 (25.7%)	45 (1.8%)	6 (0.2%)	762 (30.7%)
Sum.	194 (7.8%)	85 (3.4%)	1960(79.0%)	237(9.5%)	6 (0.2%)	2482 (100.0%)
Specific TKI in co-prescriptions	Gefitinib [16-18]	Erlotinib [19-21]	Afatinib, Apatinib, Icotinib, Anlotinib, Dasatinib, Crizotinib, Lapatinib, Regorafenib, Imatinib	Axitinib [8,22], Osimertinib [15], Nilotinib [23], Seritinib [10], Sorafenib [24],Ibrutinib [11]	Sunitinib [12,19,22]	Specific TKI in co-prescriptions

PD *=* pharmacodynamics, TKI *=* tyrosine kinase inhibitor. Significant effects: *P* < .01; potential effects: no relevant research, no hint in the package inserts; Controversy: according to the relevant literature listed.

Analysis of the effect of TKI/acid-suppressant combinations on the ADRs of the TKI showed that 73.5% of the prescriptions had potential effects, 15.0% had no effects, 11.2% had aggravating ADR symptoms but not affecting the incidence, and 0.2% had a significant effect on the incidence of ADRs (Table 5).

**Table 5:** Influence of TKI/acid-suppressant use on ADRs of TKI in 2018.

Cities	Significantly affecting the incidence of ADRs	Aggravating ADRs symptoms but not affecting the incidence	Potential effects	No effects	Sum.
Beijing	0 (0.0%)	24 (1.0%)	158 (6.4%)	20 (0.8%)	202 (8.1%)
Guangzhou	4 (0.2%)	135 (5.4%)	919 (37.0%)	163 (6.6%)	1221 (49.2%)
Hangzhou	0 (0.0%)	47 (1.9%)	215 (8.7%)	35 (1.4%)	297 (12.0%)
Zhengzhou	2 (0.1%)	73 (2.9%)	533 (21.5%)	154 (6.2%)	762 (30.7%)
Sum.	6 (0.2%)	279 (11.2%)	1825 (73.5%)	372 (15.0%)	2482 (100.0%)
Specific TKI in co-prescriptions	Crizotinib [25]	Erlotinib [21,26], Gefitinib [16,27]	Afatinib, Apatinib, Icotinib, Anlotinib, Lapatinib, Nilotinib, Regorafenib, Ibrutinib, Imatinib	Axitinib [22], Osimertinib [15], Dasatinib [4], Seritinib*, Sunitinib [22], Sorafenib [22]	Specific TKI in co-prescriptions

ADR = adverse drug reaction, TKI = tyrosine kinase inhibitor.

* According to package inserts; Significantly affecting the incidence of ADRs: *P* < .01; Potential effects: no relevant research, no hint in the package inserts.

### 3.5. Analysis of prescription source of irrational combination

We considered prescriptions with clear evidence of DDI and potential impact as irrational combination prescriptions and classified clinical departments into 3 categories according to their attributes: cancer-related internal medicine, cancer-related surgery, and noncancer-related departments. We divided the prescriptions into outpatient and inpatient prescriptions according to the patient source.

**Figure 2.**
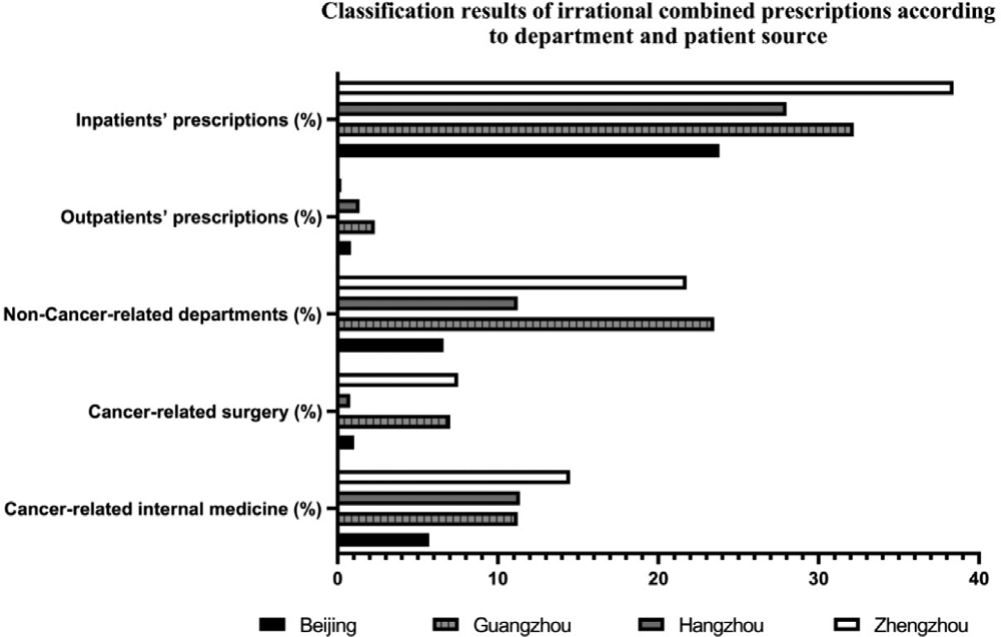
Classification results of irrational combined prescriptions according to department and patient source.

Figure 2 shows that the irrational combination rate of prescriptions (ICRP) of cancer-related surgery were low in all categories in the 4 cities, among which the highest was 7.51% in Zhengzhou, and the lowest was 0.79% in Hangzhou; ICRP of cancer-related internal medicine was the highest in all categories in Hangzhou (11.37%); ICRP of noncancer-related departments were high in Guangzhou 23.49%, Zhengzhou 21.76%, and Beijing 6.61%, while in Hangzhou it was 11.23%, slightly lower than that of cancer-related internal medicine.

The ICRP of inpatient prescriptions in the 4 cities was much higher than that of outpatients, among which the highest ICRP of inpatients was 38.41% in Zhengzhou, and the lowest was 23.82% in Beijing; the ICRP of outpatients in the 4 cities was < 3%.

## 4. Discussion

The cities included in this study are located in northern, southern, eastern, and western China. According to the latest ranking of the global healthcare access and quality index (HAQ) published by Lancet in 2016, HAQ in Beijing belonged to the category of “>91.3,” HAQ in Guangzhou and Hangzhou belonged to “82.2-91.3,” and that in Zhengzhou belonged to “74.5-82.2.”^[38]^ Regardless of the geographical distribution or HAQ, the 4 cities selected were all representative of the nation.

From 2016 to 2018, there was a significant increase in the use of TKIs in all 4 cities, including the type, prescription, quantity, and cost of TKIs used, indicating that TKIs have been increasingly used for tumor therapy in China. However, the increase in the cost of TKI used (40.40%) was lower than that in the number (71.08%), which may be related to China’s medical insurance policy in recent years: TKIs have been gradually incorporated into the national medical insurance list through price negotiation, resulting in reductions in unit prices.

Analysis of pharmacoeconomic indicators showed that regorafenib had the lowest DDC value, indicating that it had a price advantage in clinical use, whereas sunitinib had the opposite effect. The total number of medication days for various TKIs in Table 2 also reflected the impact of DDC on clinical use. The DUI data showed that there was a possibility that the doses of afatinib, icotinib, anlotinib, dasatinib, erlotinib, gefitinib, crizotinib, and regorafenib were excessive. These potential errors in medical prescribing require effective intervention from experienced physicians and pharmacists.

Based on previous studies, we found that the concomitant use of a TKI/acid-suppressant would affect the PK of the TKI to varying degrees by increasing the intragastric potential of hydrogen value or enzyme induction (inhibition), and then affect its PD or ADR, except for osimertinib. A small number of package inserts for TKIs also suggest the impact of DDI in combination with an acid-suppressant (refer to Tables 3–5 for details). We found that 36.10% of the coprescriptions had a clear impact on the PK of the TKI; 11.3% of the coprescriptions affected the PD of the TKI and 11.5% affected the ADRs of the TKI. The other coprescriptions had potential or non-influential effects.

To ensure patients safety, we considered the concomitant use of TKIs and acid-suppressants with clear or potential effects on PK, PD, and ADRs of the TKI to be irrational. Analyzing irrational combined prescriptions with prescription sources, we found that the ICRP of cancer-related surgery was significantly less than the other 2 classifications, which indicated that our rational intervention should target cancer-related internal medicine and non-cancer-related departments, especially considering the ICRP of non-cancer-related departments, which were the highest in Beijing, Guangzhou, and Zhengzhou.

Regarding the source of patients, the ICRP of inpatient prescriptions (>23% in all 4 cities) was much higher than that of outpatient prescriptions (<3% in all 4 cities). This may be related to the complete monitoring of inpatient prescription information. Meanwhile, according to our prescription sampling rules, serious prescription escape may occur in outpatients, which leads to the possibility of abundant undetected coprescriptions. The ICRP of inpatients is similar to that reported by relevant studies (24.17%),^[28]^ which also indicates that the irrational combined use of TKI/acid suppressants is a common phenomenon. Considerable attention should be paid to this and it should be corrected to avoid medical errors.

PPIs are the main acid-suppressants combined with TKIs in the various regions and are prescribed much more than H2RAs, which is also in line with the current use of acid suppressants in China (Fig. 1). It should be noted that adverse DDI was observed to a remarkable degree in PPI coadministration compared with H2RAs.^[18]^ Except for Hangzhou, use of the triple combination of TKI/PPI/H2RA existed in the 3 other cities, which could only lead to the occurrence of adverse DDI and ADRs, but it is not beneficial to the actual clinical therapy, thus requiring early intervention.

During TKI therapy, acid suppressants are always used when necessary. It is suggested that the TKI should be administered 10 h after H2RA administration or 2 h before.^[18,39,40]^ Most studies show that, due to the potent acid-suppressive effect of a PPI for 24 h, the AUC of the TKI after adjusting the administration time was not significantly different, hence the PPI was not recommended to be combined.^[18,19]^ Some studies suggested that it may be necessary to change the frequency of PPI use from twice daily to once daily while using an enteric-coated formulation, and the TKI should be administered 2 h before PPI administration.^[41]^ After the concomitant use of TKI-acid suppressant therapy, the follow-up of pharmacists should be strengthened. More attention should be paid to the plasma concentration of TKI after dose adjustment, guided by therapeutic drug monitoring, to improve the therapeutic outcomes of TKI.

In addition, changing therapeutic schedules is also an option; for example, for patients with advanced-stage EGFR-mutant lung cancer who need to use PPIs, afatinib or osimertinib may be a better choice than gefitinib.^[28]^ Interestingly, some studies found that with TKI administration combined with acidic beverages (such as cola and soda), betaine HCl may lead to temporary gastric re-acidification, which may facilitate the dissolution, solubilization, and absorption of the TKI within the critical absorption window.^[16,17,28,42]^

In recent years, the development of TKIs is one of the main driving factors of tumor therapeutics, followed by an obvious related adverse event “financial toxicity.”^[43–46]^ The high unit price of TKIs leads to a huge economic burden on patients. Long-term, even life-long, medication leads to the fact that even if many TKIs have been included in medical insurance, it can only alleviate the burden but not eliminate the economic problems of patients.^[47,48]^ Therefore, the rational and effective use of TKIs is particularly important for the efficacy and economic aspects of patients, and it is also the goal of hospital doctors and pharmacists.

## 5. Conclusion

With the increasing use of TKIs, the proportion of irrational combinations of TKI/acid-suppressants has also increased significantly, which is a nationwide phenomenon which urgently needs to be addressed. This data analysis of TKI-related use in Grade-III class-A hospitals in 4 representative cities in China provides a valuable basis for rational follow-up intervention. With active and effective intervention from doctors and pharmacists, the rationality of TKI therapy can be improved, and patients will benefit from it.

## Acknowledgments

We acknowledge the Hospital Prescription Cooperation Project of China for collecting and providing the data used in this study. We also acknowledge the efforts of the Zhejiang Pharmaceutical Association Hospital Pharmacy Management Foundation. We would like to thank Editage (www.editage.cn) for English language editing.

## Author contributions

**Conceptualization:** Fangting Chen, Wendong Yao, Jianping Wang, Zheng Shi

**Data curation:** Fan Wu, Rui Xie.

**Editing:** Wendong Yao, Rui Xie.

**Formal analysis:** Fangting Chen.

**Funding acquisition:** Zheng Shi.

**Methodology:** Zheng Shi.

**Resources:** Fan Wu, Jianping Wang.

**Reviewing and editing:** Jianping Wang, Zheng Shi.

**Writing:** Fangting Chen.
